# Effect of Dapagliflozin in Patients with Heart Failure: A Systematic Review and Meta-Analysis

**DOI:** 10.5334/gh.1258

**Published:** 2023-08-22

**Authors:** Ahmed E. Ali, Muhammad Sabry Mazroua, Mariam ElSaban, Nadia Najam, Aditi S. Kothari, Taha Mansoor, Tanya Amal, Joanna Lee, Rahul Kashyap

**Affiliations:** 1 Mansoura Specialized Hospital, Mansoura, Egypt; 2 Global Remote Research Scholars Program, St. Paul, Minnesota, USA; 3 Mansoura University Hospitals, Mansoura, Egypt; 4 Department of Anesthesiology & Perioperative Medicine, Mayo Clinic, Rochester, Minnesota, USA; 5 Hamdard College of Medicine & Dentistry, India; 6 BJ Medical College, Ahmedabad, India; 7 Shifa College of Medicine, Islamabad, Pakistan; 8 Maulana Azad Medical College, New Delhi, India; 9 David Tvildiani Medical University, Tbilisi, Georgia; 10 Department of Critical Care Medicine, Mayo Clinic, Rochester, Minnesota, USA; 11 Medical Director of Research, WellSpan Health, York, Pennsylvania, USA

**Keywords:** Dapagliflozin, Forxiga, SGLT2-inhibitors, Heart failure, Diabetes Mellitus

## Abstract

**Background:**

Heart failure (HF) is a major cause of recurrent hospitalization and death worldwide. Sodium-glucose cotransporter-2 inhibitors including dapagliflozin are anti-diabetic drugs with promising cardiovascular (CV) effects. We performed systematic review and meta-analysis of randomized controlled trials investigating the effects of dapagliflozin in heart failure patients.

**Methods:**

We searched PubMed, Scopus and ScienceDirect databases. A total of 1,567 studies from January 2017 to September 10, 2022, were screened. After applying exclusion criteria, 22 studies were retrieved for full-text screening, and nine of them were eligible for this meta-analysis. Effect estimates for dichotomous variables were expressed as risk ratio (RR) and 95% CI. The primary outcomes were the incidence of all-cause mortality, hospitalization due to HF, and CV death. This review was registered on PROSPERO with ID CRD42022347793.

**Results:**

A total of 14,032 patients were included. The overall risk ratio of all-cause mortality favored the dapagliflozin group over the placebo/standard therapy group (RR = 0.89, 95% CI: 0.82–0.97, P = 0.006) and the pooled studies were not heterogenous (I^2^ = 0%). Additionally, dapagliflozin significantly reduced the hospitalization due to heart failure (RR = 0.76, 95% CI: 0.70–0.84, P > 0.00001, I^2^ = 0%), cardiovascular death (RR = 0.87, 95% CI: 0.78–0.97, P = 0.01, I^2^ = 0%) and their composite outcomes.

**Conclusion:**

Dapagliflozin reduces the risk of all-cause mortality, heart failure hospitalizations and cardiovascular death in a wide range of heart failure patients.

## Introduction

Heart failure (HF) is a major cause of recurrent hospitalization and death worldwide despite established therapy [1]. Type 2 diabetes mellitus (T2DM) is also a risk factor for developing HF [2] and HF-associated complications such as death [3]. One class of antidiabetic drugs, sodium-glucose transporter 2 (SGLT-2) inhibitors, have been shown by four large-scale clinical trials involving T2DM patients that they reduce the risk of hospitalization for heart failure [4,5,6,7]. These trials did not have patients with heart failure at baseline, so the outcomes only reflected the prevention of heart failure. Since then, many randomized controlled clinical trials (DELIVER, EMPEROR-Preserved trials, DAPA-HF, EMPEROR-Reduced trial, DEFINE-HF, and SOLOIST-WHF trial) have been performed to evaluate the efficacy of SGLT-2 inhibitors in reducing major adverse outcomes in patients with established heart failure, regardless of the presence of T2DM [8,9,10,11,12,13]. Based on these results, the ACC/AHA/HFSA incorporated SGLT-2 inhibitors as recommended agents across the spectrum of HF [14]. Previously published meta-analyses concentrated on all SGLT-2 inhibitors further solidified the support for establishing SGLT-2 inhibitors as a foundational therapy for heart failure [15,16,17,18]. In this systematic review and meta-analysis, we pre-specified to examine the effect of dapagliflozin in comparison to placebo or standard HF therapy on all-cause mortality, HF hospitalizations, and CV death in HF patients.

## Methods

### Search strategy and inclusion criteria

**Figure 1:**
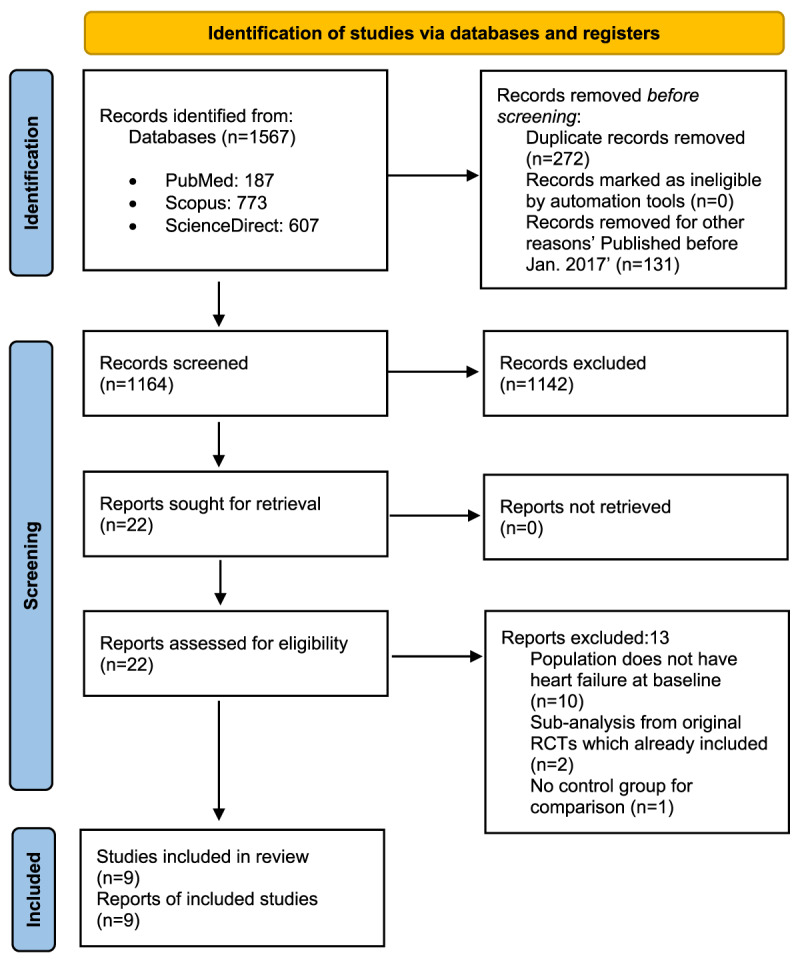
PRISMA Flow Chart of Dapagliflozin in Patients with Heart Failure.

This meta-analysis was conducted while adhering to the Preferred Reporting Items for Systematic Reviews and Meta-Analyses (PRISMA) guidelines [19]. The protocol of this systematic review was registered on the International Prospective Register of Systematic Reviews (PROSPERO ID: CRD42022347793). A systematic literature search was conducted for randomized trials exploring the cardiovascular and heart failure outcomes in patients with type 2 diabetes utilizing the SGLT2 Inhibitor: Dapagliflozin. The search strategy and keywords are available in Supplementary data. Multiple databases were queried, including PubMed, Scopus, and ScienceDirect, from January 1, 2017, until September 10, 2022. The search was restricted to articles in the English Language and if free full text was available. A total of 1,567 eligible studies were identified. Abstract and full-text screenings were performed by two reviewers and were done utilizing the Rayyan software. Discrepancies in the inclusion decision were resolved by a third author. Articles were included if they were randomized control trials (RCT) exploring outcomes in patients utilizing dapagliflozin compared to placebo or standard therapy among adult patients (≥18 years old) with diagnosed heart failure. Heart failure, for the purpose of this review, was defined as a NYHA class ≥1. Articles were excluded if they were observational studies, case series, case reports, reviews, or only protocols of RCTs. The complete PRISMA flow chart of articles’ inclusion was depicted in Figure 1.

### Data extraction

Included articles were thoroughly read by two reviewers to extract study characteristics (First author, year of publication, study arms and populations, drug interventions, control group definition, and the outcomes explored) and patients’ characteristics (Patients with HF at baseline, average Age, percent females, breakdown of NYHA classification, prior CV medications, presence of IHD and DM, baseline LVEF and baseline BNP). The primary outcomes included all-cause mortality, worsening heart failure episodes (hospitalization or the equivalent, i.e., an urgent heart failure visit), and cardiovascular mortality. Secondary outcomes included composite outcomes that include any of the primary outcomes.

### Quality assessment

Two independent reviewers assessed the quality of the included RCTs using the Modified Cochrane Risk of Bias Assessment Tool [20]. This tool explored the following domains of trials’ methodological quality including random sequence generation, allocation concealment, blinding of participants and personnel, blinding of outcomes assessment, attrition bias due to missing data, reporting bias, and other sources of bias [20]. Each of the aforementioned domains received a grade of low risk, high risk, or unclear risk. Further, publication bias was assessed via funnel plot asymmetry.

### Statistical analysis

The statistical analysis was performed using Review Manager software (RevMan) version 5.3. Outcomes were analyzed if they were reported in two or more studies. Effect estimates were analyzed as mean differences and relative risks utilizing the fixed effect model and inverse variance method. The leave-one-out test for sensitivity analysis was performed. Measures of heterogeneity included the I^2^ and Chi^2^ indices. A level higher than 50% was defined as high heterogeneity. A subgroup analysis, if two or more studies were reported per subgroup arm, was conducted for relevant outcomes based on patients’ age groups or diabetic status. A two-sided p-value of <0.05 was considered significant.

## Results

### Eligible studies

A total of 1,567 studies were identified through databases. Duplicates were removed and the screening process was done. A total of 22 articles were retrieved for full-text screening. Finally, nine RCTs were included in this systematic review representing a total of 14,032 participants (7,007 patients in the dapagliflozin group and 7,025 patients in the control group).

### Studies and population characteristics

Seven studies included patients with heart failure as the original sample population [8,10,12,21,22,23,24], and two studies [6,25] included patients with CKD or Type 2 diabetes as the original population, but they were included as they had a prespecified analysis of outcome according to the presence of heart failure at baseline or not. The primary outcomes were composite outcomes including all-cause mortality, cardiovascular death, hospitalization due to heart failure, and emergency room visits due to heart failure. A summary of the included studies consisting of the first author, year of publication, population, control group, intervention group, outcomes, and source of funding is available in Table 1.

**Table 1:** Summary of the Included Studies.


STUDY	POPULATION	INTERVENTION	CONTROL	OUTCOMES	SOURCE OF FUNDING

Ibrahim 2020	Patients with T2DM admitted with decompensated HFrEF	Dapagliflozin 10 mg with recommended therapy.	Recommended therapy	Weight loss and dyspnea improvement.	The authors reported receiving no fund for this study

McMurray 2019	Patients with HFrEF	Dapagliflozin (10 mg once daily) with recommended therapy.	Placebo with recommended therapy.	The primary outcome: a composite of worsening HF or CV death. The secondary outcomes: 1) a composite of hospitalization for HF or CV death; 2) the total number of hospitalizations for HF and CV deaths; 3) the change in the total symptom score on the KCCQ; 4) a composite of worsening renal function; and 5) death from any cause.	AstraZeneca

McMurray 2021	Patients with CKD, with and without type 2 diabetes, with or without HF.	Dapagliflozin 10 mg	Placebo	The primary outcome: a composite of the time to the first occurrence of any of the following: 50% decline in eGFR, onset of ESKD, or kidney/CV death. Secondary outcomes were: 1) a kidney composite outcome (primary endpoint minus CV death); 2) a CV composite outcome consisting of HF hospitalization or death from CV causes; 3) death from any cause; 4) time-to-first HF hospitalization; and 5) total number of HF hospitalizations.	AstraZeneca

Nassif 2019	Patients with HFrEF	Dapagliflozin 10 mg daily in addition to guideline directed standard of care therapy	Placebo in addition to guideline directed standard of care therapy	Primary end points were (1) Mean NT-proBNP and (2) a composite of the proportion of patients that achieved a meaningful improvement in health status (≥5-point increase in KCCQ-OS) or (≥20% decrease in NT-proBNP). Key secondary end points included proportion of patients with meaningful change in KCCQ, and NT-proBNP at each time point, mean BNP and proportion of patients with meaningful change in BNP, functional status based on 6-minute walk test, change in weight, systolic BP and HbA1c. Exploratory end points included a composite of hospitalization for HF or urgent HF visits.	AstraZeneca

Nassif 2021	Patients with HFpEF	Dapagliflozin	Placebo.	Primary endpoint is change in KCCQ-CS. Secondary endpoints included the 6MWT, KCCQ-OS, clinically meaningful changes in KCCQ-CS and -OS, and changes in weight, natriuretic peptides, glycated hemoglobin and systolic blood pressure Exploratory clinical endpoints: HF hospitalizations or urgent HF visits.	AstraZeneca

Palau 2022	Stable patients with HFrEF	Dapagliflozin	Placebo	The primary outcome: a change in peakVO2 at 1 and 3 months. The secondary outcomes: 1) changes at 1 and 3 months in 6MWT distance; 2) quality of life (MLHFQ); and 3) echocardiographic parameters (diastolic function, left chamber volumes, and left ventricular EF).	Grant from AstraZeneca, Clinical Research and Clinical Trials Unit including Health Research Institute, Spanish Clinical Research Network, and CIBER Cardiovascular

Singh 2020	Patients with T2DM and HFrEF	Dapagliflozin 10 mg daily plus usual therapy	Placebo plus usual therapy	The primary outcome: change in LVESV. The secondary outcomes: 1) LVEDV; 2) LVMI; 3) LVEF; and 4) a range of clinical and biochemical markers of HF.	Grant from the European Foundation for the Study of Diabetes, Clinical Diabetes Research Program in Macrovascular Complications of Diabetes. Other support by National Health Service Education for Scotland/Chief Scientist Office Post- Doctoral Clinical Lectureship and the British Heart Foundation

Solomon 2022	Patients with HFpEF	Dapagliflozin (10 mg once daily) plus usual therapy	Placebo plus usual therapy	The primary outcome: a composite of worsening HF or CV death. The Secondary outcomes: 1) the total number of worsening HF events and CV deaths; 2) the change in the total symptom score on the KCCQ; 3) CV death; and 4) death from any cause.	AstraZeneca

Wiviott 2019	Patients with T2DM who had or were at risk for ASCVD.	Dapagliflozin	Placebo	The primary safety outcome was a composite of major adverse cardiovascular events, defined as CV death, MI, or ischemic stroke. The primary efficacy outcomes were MACE, and a composite of CV death or hospitalization for HF. The secondary efficacy outcomes were a renal composite (≥40% decrease in eGFR to <60 ml/minute/1.73 m^2^ of body surface area, new ESKD, or death from renal or CV causes) and death from any cause.	AstraZeneca and Bristol-Myers Squibb


The trials that were included in this review were published before September 10, 2022. The mean/median age ranged from 60 to 72 years. In all included trials, the male sex proportion was higher than 50% except Nassif 2021 study [21]. Seven studies included exclusively patients with HF. NYHA II group was the most predominant HF classification among studies. Baseline mean/median LVEF ranged from 25.7 to 60%. Baseline mean/median NT-proBNP ranged from 641 to 1,620 pg/ml. The most frequent previous therapies in most of the included trials were ACEIs, ARBs, MRAs, Digitalis, and diuretics. Baseline characteristics and detailed information about the included studies are summarized in Table 2.

**Table 2:** Baseline Characteristics of the Patients in the Included Studies.


STUDY	STUDY GROUPS (N)	HF AT BASELINE	AGE	FEMALE (%)	NYHA (%)	IHD (%)	DM (%)	BASELINE LVEF	BASELINE NT PROBNP PG/ML	PRIOR CV THERAPY (%)

Ibrahim 2020	Dapagliflozin + Standard therapy (50)	50	62.02 ± 8.8	22 (44)	NR	NR	50 (100)	32.54 ± 2.99	NR	ACE-I: 37 (74) ARBs: 9 (18) ARNI: 4 (8) BBs: 3 (6) Digitalis: 18 (36) MRAs: 41 (82) Thiazide: 5 (10)

	Standard therapy (50)	50	60.64 ± 9.9	24 (48)	NR	NR	50 (100)	32.23 ± 2.49	NR	ACE-I: 33 (66) ARBs: 12 (24) ARNI: 2 (4) BBs: 2 (4) Digitalis: 20 (40) MRAs: 43 (86) Thiazide: 4 (8)

McMurray 2019	Dapagliflozin (2373)	2373	66.2 ± 11.0	564 (23.8)	II: 1606 (67.7) III: 747 (31.5) IV: 20 (0.8)	1316 (55.5)	993 (41.8)	31.2± 6.7	1428 (857–2655)	ACE-I: 1332 (56.1) ARBs: 675 (28.4) ARNI: 250 (10.5) BBs: 2278 (96.0) Digitalis: 445 (18.8) MRAs: 1696 (71.5) Diuretic: 2216 (93.4)

	Placebo (2371)	2371	66.5 ± 10.8	545 (23.0)	II: 1597 (67.4) III: 751 (31.7) IV: 23 (1.0)	1358 (57.3)	990 (41.8)	30.9± 6.9	1446 (857–2641)	ACE-I: 1329 (56.1) ARBs: 632 (26.7) ARNI: 258 (10.9) BBs: 2280 (96.2) Digitalis: 442 (18.6) MRAs: 1674 (70.6) Diuretic: 2217 (93.5)

McMurray 2021*	Dapagliflozin (2152)	235 (10.9%)	61.8± 12.1	709 (32.9)	NR	NR	1455 (67.6)	NR	NR	ACE-I: 673 (31.3) ARBs: 1444 (67.1) Diuretic: 928 (43.1) Statin: 1395 (64.8)

	Placebo (2152)	233 (10.8%)	61.9± 12.1	716 (33.3)	NR	NR	1451 (67.4)	NR	NR	ACE-I: 681 (31.6) ARBs: 1426 (66.3) Diuretic: 954 (44.3) Statin: 1399 (65.0)

Nassif 2019	Dapagliflozin (131)	131	62.2 ± 11.0	36 (27.5)	II: 91 (69.5) III: 40 (30.5)	70 (53.4%)	81 (61.8)	27.2±8.0	1136 (668, 2465)	ACEI/ARB: 76 (58.0) ARNI: 47 (35.9) BBs: 130 (99.2) Hydralazine: 19 (14.5) Long-acting nitrates: 17 (13.0) MRA: 76 (58.0) Loop diuretics: 114 (87.0) Digoxin: 25 (19.1) Lipid-lowering agents: 107 (81.7) Anticoagulant agent: 58 (44.3)

	Placebo (132)	132	60.4 ± 12.0	34 (25.8)	II: 82 (62.1) III: 50 (37.9)	69 (52.3%)	85 (64.4)	25.7±8.2	1136 (545, 2049)	ACEI/ARB: 80 (60.6) ARNI: 38 (28.8) BBs: 124 (93.9) Hydralazine: 26 (19.7) Long-acting nitrates: 22 (16.7) MRA: 84 (63.6) Loop diuretics: 111 (84.1) Digoxin: 21 (15.9) Lipid-lowering agents: 104 (78.8) Anticoagulant agent: 42 (31.8)

Nassif 2021	Dapagliflozin (162)	162	69 (64, 77)	92 (56.8)	II: 96 (59.3) III/IV: 65 (40.1)	32 (19.8)	90 (55.6)	60 (55, 65)	641 (373, 1210)	ACEI/ARB: 98 (60.5%) ARNI: 2 (1.2%) BBs: 119 (73.5%) Hydralazine: 25 (15.4%) Long-acting nitrates: 34 (21.0%) MRA: 50 (30.9%) Loop diuretics: 151 (93.2%) Lipid-lowering agents: 132 (81.5%) Anticoagulant agent: 71 (43.8%)

	Placebo (162)	162	71 (63, 78)	92 (56.8)	II: 90 (55.6) III/IV: 72 (44.4)	31 (19.1)	91 (56.2)	60 (54, 65)	710 (329, 1449)	ACEI/ARB: 98 (60.5) ARNI: 3 (1.9) BBs: 116 (71.6) Hydralazine: 18 (11.1) Long-acting nitrates: 27 (16.7) MRA: 68 (42.0) Loop diuretics: 135 (83.3) Lipid-lowering agents: 127 (78.4) Anticoagulant agent: 84 (51.9)

Palau 2022	Dapagliflozin (45)	45	69.8 (62.4–74.0)	10 (22.2)	II/IV: 41 (91.1)	27 (60.0)	16 (35.6)	33.7± 5.3	1085 (889–2100)	ACEI or ARB or sacubitril/valsartan: 44 (97.8) Sacubitril/valsartan: 40 (88.9) BBs: 41 (91.1) MRA: 35 (77.8) Loop diuretics: 39 (86.7)

	Placebo (45)	45	67.3 (60.8–75.1)	11 (24.4)	II/IV: 39 (86.7)	22 (48.9)	13 (28.9)	34 ± 5.3	1620 (889–2328)	ACEI or ARB or sacubitril/valsartan: 43 (95.6) Sacubitril/valsartan: 40 (88.9) BBs: 41 (91.1) MRA: 32 (71.1) Loop diuretics: 38 (84.4)

Singh 2020	Dapagliflozin (28)	28	66.9 ± 7.0	10 (35.7)	I: 12 (42.9) II: 13 (46.4) III: 3 (10.7)	15 (53.6)	28 (100)	44.5 ± 12.4	NR	ACEI/ARB: 25 (89.3) BBs: 24 (85.7) MRA: 13 (46.4)

	Placebo (28)	28	67.4 ± 6.8	9 (32.1)	I: 13 (46.4) II: 11 (39.3) III: 4 (14.3)	15 (53.6)	28 (100)	46.5 ± 11.7	NR	ACEI/ARB: 25 (89.3) BBs: 22 (78.6) MRA: 10 (35.7)

Solomon 2022	Dapagliflozin (3131)	3131	71.8 ± 9.6	1364 (43.6)	II: 2314 (73.9) III: 807 (25.8) IV: 10 (0.3)	NR	1401 (44.7)	54.0 ± 8.6	AF: 1408 (956, 2256) No AF: 729 (472, 1299)	ACE-I: 1144 (36.5) ARBs: 1133 (36.2) ARNI: 165 (5.3) BBs: 2592 (82.8) MRAs: 1340 (42.8) Loop Diuretic: 2403 (76.7)

	Placebo (3132)	3132	71.5 ± 9.5	1383 (44.2)	II: 2399 (76.6) III: 724 (23.1) IV: 8 (0.3)	NR	1405 (44.9)	54.3 ± 8.9	AF: 1387 (965.5, 2180.5) No AF: 704 (467, 1265)	ACE-I: 1151 (36.7) ARBs: 1139 (36.4) ARNI: 136 (4.3) BBs: 2585 (82.5) MRAs: 1327 (42.4) Loop Diuretic: 2408 (76.9)

Wiviott 2019	Dapagliflozin (8582)	852 (9.9%)	63.9 ± 6.8	3171 (36.9)	NR	2824 (32.9)	8582 (100)	NR	NR	ACEI/ARB: 6977 (81.3) BBs: 4498 (52.4) Diuretic: 3488 (40.6) Anti-platelet agents: 5245 (61.1) Statin or ezetimibe: 6432 (74.9)

	Placebo (8578)	872 (10.2%)	64.0 ± 6.8	3251 (37.9)	NR	2834 (33.0)	8578 (100)	NR	NR	ACEI/ARB: 6973 (81.3) BBs: 4532 (52.8) Diuretic: 3479 (40.6) Anti-platelet agents: 5242 (61.1) Statin or ezetimibe: 6436 (75.0)


* Baseline characteristics of McMurray 2021 study were reported from the original clinical trial data [33].

### Risk of bias assessment

**Figure 2:**
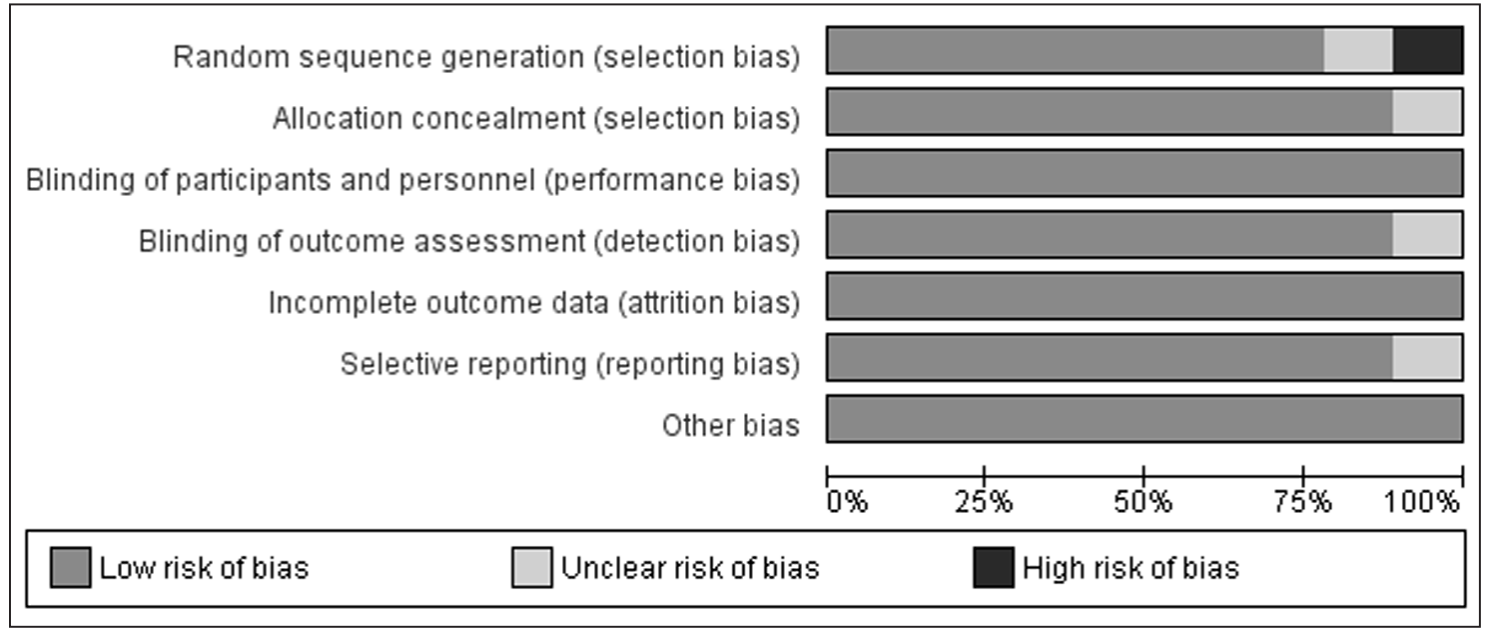
Risk of Bias Graph.

The overall possibility of bias in the selected reports and other biases was low. Six out of nine RCTs had an overall low risk of bias. Three studies presented some concerns as shown in the risk of bias graph (Figure 2). The risk of bias summary is available in Supplementary data (Figure 1). The publication bias was assessed using a funnel plot which showed a low risk of publication bias as shown in Supplementary Figure 2.

### Meta-analysis

**Figure 3:**
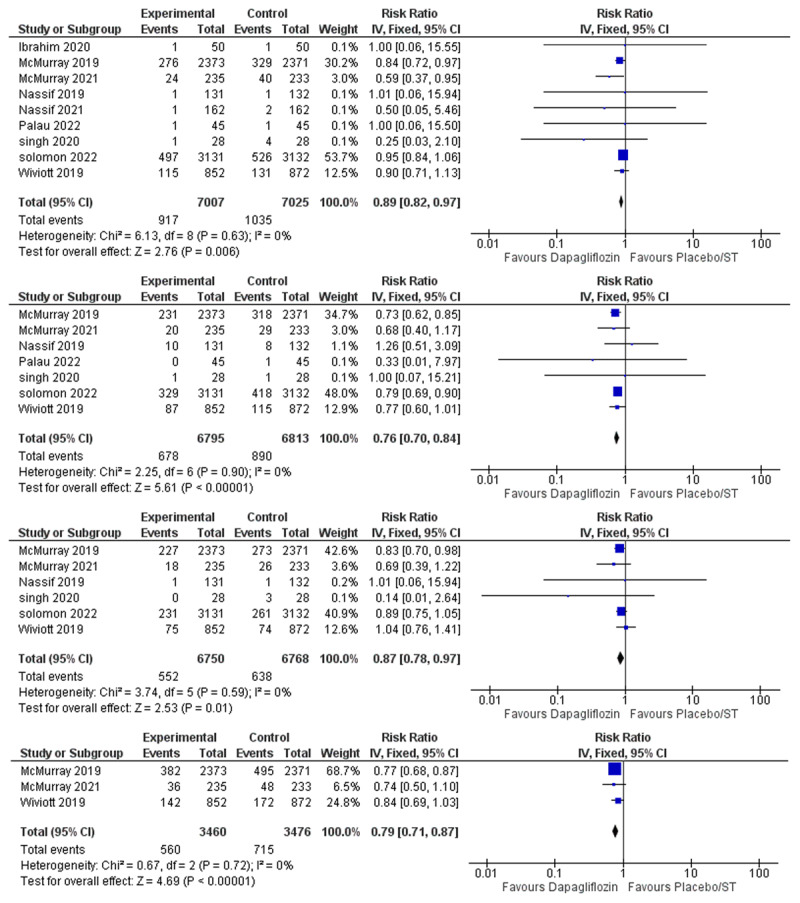
Forest Plot of **A)** All-Cause Mortality. **B)** Heart Failure Hospitalizations. **C)** Cardiovascular Deaths. **D)** Cardiovascular Deaths or Heart Failure Hospitalizations.

**Figure 4:**
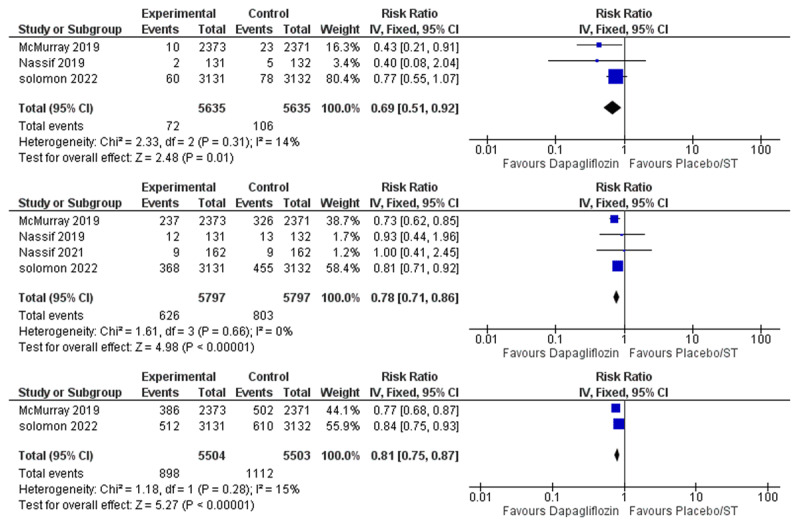
Forest Plot of **A)** Urgent Heart Failure Visits. **B)** Heart Failure Hospitalization or Urgent Heart Failure Visit. **C)** Worsening Heart Failure Event (hospitalization or urgent visit) or Cardiovascular Death.

- Effect of dapagliflozin on all-cause mortality:
  The meta-analysis of the included nine studies showed that the overall risk ratio of all-cause mortality favored the dapagliflozin group over the placebo/standard therapy group (RR = 0.89, 95% CI: 0.82–0.97, P = 0.006), and the pooled studies were not heterogenous (I^2^ = 0%) Figure 3(A).
- Effect of dapagliflozin on heart failure hospitalization:
  Seven studies reported heart failure hospitalization. Dapagliflozin was found to reduce heart failure hospitalization (RR = 0.76, 95% CI: 0.70–0.84, P > 0.001), and no statistical heterogeneity was found among the included studies (I^2^ = 0%) Figure 3(B).
- Effect of dapagliflozin on cardiovascular death:
  Six studies were included in the meta-analysis. Dapagliflozin was proven to reduce cardiovascular death (RR = 0.87, 95% CI: 0.78–0.97, P = 0.01), and the pooled studies were not heterogenous (I^2^ = 0%) Figure 3(C).
- Effect of dapagliflozin on cardiovascular deaths or heart failure hospitalizations:
  Three studies reported the composite outcome of CV death or HF hospitalization. Dapagliflozin decreased the incidence of CV death/HF hospitalization (RR = 0.79, 95% CI: 0.71–0.87, P > 0.001), and no statistical heterogeneity was found (I^2^ = 0%) Figure 3(D).
- Effect of dapagliflozin on urgent heart failure visits:
  Three of the included studies reported urgent emergency room visits due to heart failure as an outcome. Dapagliflozin was found to reduce the incidence of urgent heart failure visits (RR = 0.69, 95% CI: 0.51–0.92, P > 0.01), and no significant heterogeneity was found among studies (I^2^ = 14%) Figure 4(A).
- Effect of dapagliflozin on heart failure hospitalization or urgent heart failure visits:
  Four studies were included in the meta-analysis. The overall risk ratio of HF hospitalization/Urgent HF visits favored the dapagliflozin group over the placebo/standard therapy group (RR = 0.78, 95% CI: 0.71–0.86, P > 0.001), and there was no heterogeneity among the included studies (I^2^ = 0%) Figure 4(B).
- Effect of dapagliflozin on worsening heart failure event (hospitalization or urgent visit) or Cardiovascular Death:
  Two studies were eligible for the meta-analysis. Dapagliflozin reduced the incidence of the composite outcome of worsening heart failure event/cardiovascular death (RR = 0.81, 95% CI: 0.75–0.87, P > P > 0.001), and no significant heterogeneity was found among studies ((I^2^ = 15%) Figure 4(C).

### Subgroup analysis according to the type of HF

**Figure 5:**
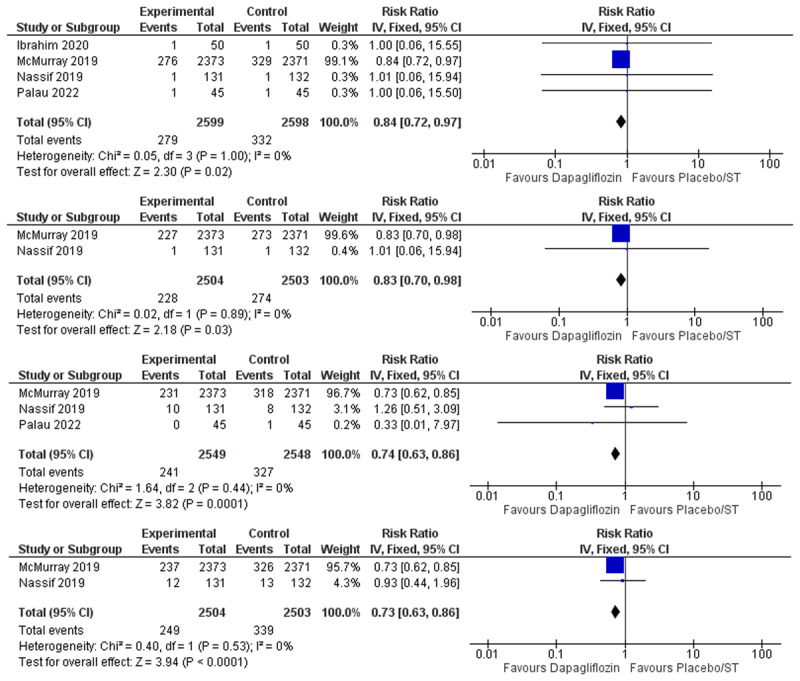
HFrEF Subgroup Forest Plots of **A)** All-Cause Mortality. **B)** Cardiovascular Deaths. **C)** Heart Failure Hospitalizations. **D)** Heart Failure hospitalization or Urgent Heart Failure Visit.

**Figure 6:**
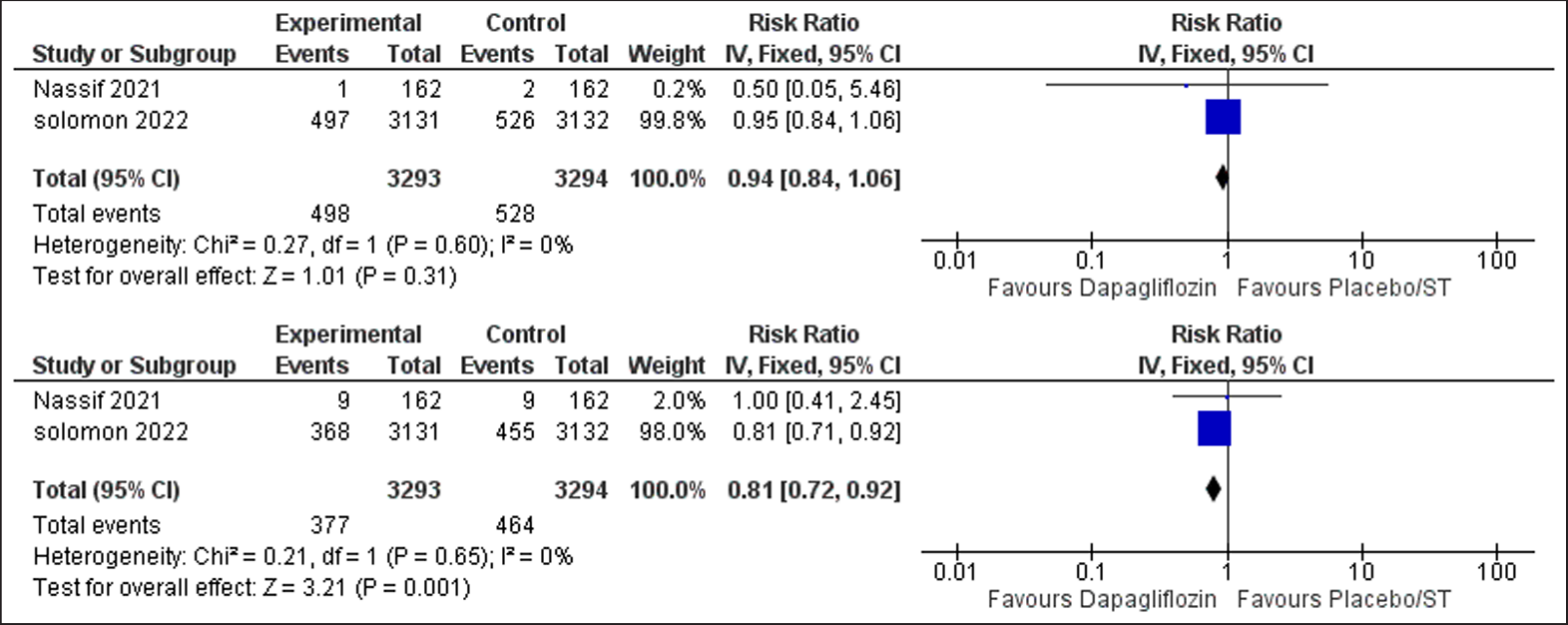
HFpEF Subgroup Forest Plots of **A)** All-Cause Mortality. **B)** Heart Failure Hospitalization or Urgent Heart Failure Visit.

We performed subgroup analysis based on the type of HF, either HFrEF or HFpEF. Four studies included exclusively patients with HFrEF [10,12,22,24] and two studies included patients with HFpEF [8,21]. In the HFrEF subgroup, the overall risk ratio favored the dapagliflozin group over the placebo/standard therapy group for all-cause mortality (RR = 0.84, 95% CI: 0.72–0.97, P = 0.02), CV death (RR = 0.83, 95% CI: 0.70–0.98, P = 0.03), HF hospitalization (RR = 0.74, 95% CI: 0.63–0.86, P = 0.0001) and the composite outcome of HF hospitalization or urgent HF visits (RR = 0.73, 95% CI: 0.63–0.86, P >0.0001). The pooled studies were not heterogenous (I2 = 0%) Figure 5. Whereas in the HFpEF subgroup, there was no statistically significant difference between the dapagliflozin group and the placebo/standard therapy group for all-cause mortality (RR = 0.94, 95% CI: 0.84–1.06, P = 0.31), while dapagliflozin was found to reduce the incidence of the composite outcome of HF hospitalization or urgent HF visits (RR = 0.81, 95% CI: 0.72–0.92, P = 0.001) and no heterogeneity was found (I2 = 0%) Figure 6.

## Discussion

In this study, which was designed to study the effect of dapagliflozin on patients with heart failure, we found a statistically significant association with a reduction in all-cause mortality, cardiovascular mortality, and heart failure-related hospitalization. Additionally, an improvement in the KCCQ score, 6MWT results, NT-pro BNP levels, echocardiographic measures, change in weight, and urgent heart failure visits were observed with dapagliflozin compared to placebo. The results were consistent across the included studies and no statistical heterogeneity was found in all outcomes analysis with I^2^ value between 0 and 15%.

The meta-analysis included patients with and without diabetes mellitus and hence it can be extrapolated that the effect of dapagliflozin in heart failure is independent of its effect in diabetics and the drug can be used in patients with heart failure who do not have diabetes. Additionally, we conducted a subgroup analysis based on the type of heart failure in the included studies. Results from both HFrEF and HFpEF subgroups demonstrated that dapagliflozin had a superior effect compared to placebo in reducing the occurrence of the composite outcome of HF hospitalization or urgent HF visits. Although the difference in all-cause mortality incidence was statistically insignificant between dapagliflozin and placebo in the HFpEF subgroup (P = 0.31), this may be explained by the limited number of studies (two studies) that included only HFpEF patients in our meta-analysis [8,21]. Hence, further studies investigating the effects of dapagliflozin in patients with HFpEF are warranted. The average baseline LVEF in our study was as low as 27.2 in the dapagliflozin group and 25.7 in the placebo group.

The mechanism by which dapagliflozin works in heart failure patients is by acting on the SGLT-2 receptors in the kidney leading to osmotic diuresis of glucose along with natriuresis and loss of water thereby decreasing the preload on the heart [26]. Another possible mechanism is its effect on afterload via its action on the endothelium. This occurs mainly by a decrease in vascular stiffness thereby reducing the afterload [26]. SGLT2 inhibitors also increase cardiac output by optimizing myocardial substrate use by reducing the cardiac uptake of carbohydrates and increasing the uptake of B-hydroxybutyrate and ketone bodies [27]. This class of drugs is anti-inflammatory and hence also inhibits myocardial fibrosis [28]. A systematic review by Cai et al [27] shows that the beneficial effects of dapagliflozin are not dependent on the left ventricular ejection fraction and hence dapagliflozin is suitable for both patients with HFrEF and HFpEF. Whereas a meta-analysis by Zhai et al. [29] showed that dapagliflozin did not show any superior benefit over placebo in terms of cardiovascular death and all-cause mortality in the HFpEF subgroup.

In clinical practice, dapagliflozin is associated with several adverse effects like-Mycotic genital tract infections (vulvovaginitis or balanitis) and urinary tract infections [30]. Other side effects associated with dapagliflozin are hypotension, hypoglycemia, and lower serum uric acid levels [31]. The drug is also associated with several laboratory parameters changes such as an increase in LDL-cholesterol levels, hematocrit, and phosphorus levels, and it is important to monitor their levels before starting treatment [30]. SGLT2 inhibitors have been associated with diabetic ketoacidosis (DKA), and the DAPA-HF trial showed that all cases of DKA occurred in patients with type-2 DM and that the incidence of DKA was 0.1% with dapagliflozin and 0% with placebo [32].

### Strengths

In this meta-analysis, we have several strengths. We included all clinical trials that have patients with heart failure as the original study population or have pre-specified analysis to their population randomization according to the presence of heart failure at baseline or not. This includes a large clinical trial that was published in late 2022 on patients with HFpEF and the results were nearly the same when compared to HFrEF patients; this finding confirms the effectiveness and safety of dapagliflozin in patients with HFpEF which was questionable in many previous studies.

### Limitations

This meta-analysis only included clinical trials studying the effects of dapagliflozin and therefore there is a lack of information about the effects of other SGLT-2 inhibitors in patients with cardiac failure. Also, there were some missing data regarding baseline patients’ characteristics in a few of the included studies. several of the included studies have a small sample size; the generalizability of these results could be debated.

## Conclusion

In a comprehensive systematic review, dapagliflozin has shown an association with reducing the incidence of All-cause mortality, cardiovascular death, hospitalization due to heart failure, and their composite outcomes in patients with heart failure. These results were statistically significant in this patient population whether they have diabetes mellitus or not at the baseline. Therefore, dapagliflozin could be used effectively in heart failure patients with or without diabetes mellitus.

## Additional File

The additional file for this article can be found as follows:

10.5334/gh.1258.s1 Supplementary File. The supplementary file contains the search strategy and keywords that were used for this study, the risk of bias summary, and the funnel plot for assessment of the publication bias.

## Abbreviations

HF: Heart failure

CV: Cardiovascular

RR: Risk ratio

CI: Confidence interval

T2DM: Type 2 diabetes mellitus

SGLT-2: Sodium-glucose transporter 2

RCT: Randomized control trials

NYHA: New York Heart Association

LVEF: Left ventricular ejection fraction

IHD: Ischemic heart disease

MI: Myocardial infarction

MACE: Major adverse cardiovascular events

NT proBNP: N-terminal pro-B-type natriuretic peptide

CKD: Chronic kidney disease

ACEIs: Angiotensin-converting enzyme inhibitors

ARBS: Angiotensin receptor blockers

ARNIs: Angiotensin receptor/neprilysin inhibitors

MRAs: Mineralocorticoid receptor antagonists

BBs: Beta-blockers

KCCQ: The Kansas City Cardiomyopathy Questionnaire

6MWT: 6-minute walk test

HFrEF: Heart failure and reduced ejection fraction

HFpEF: Heart failure and preserved ejection fraction

DAPA: Dapagliflozin

ST: Standard therapy

DKA: Diabetic ketoacidosis

eGFR: Estimated glomerular filtration rate

ESKD: End-stage kidney disease

LVESV: Left ventricular end-systolic volume

LVMI: Left ventricular mass index

MLHFQ: The Minnesota Living with Heart Failure Questionnaire

NR: Not reported
