# Supplementary material for: Effect of Dapagliflozin in Patients with Heart Failure: A Systematic Review and Meta-Analysis
Source: Glob Heart. 2023 Aug 22;18(1):45. doi: 10.5334/gh.1258 (PMC10453961; doi:10.5334/gh.1258)

Contents:

1- **Search Strategy and Keywords**

2- **Figure 1: Risk of bias summary.**

3- **Figure 2: Funnel plot for publication bias assessment.**

Search Strategy and Keywords

1) PubMed

("dapagliflozin"[Mesh] OR "dapagliflozin"[Supplementary Concept] OR “dapagliflozin”[tiab] OR "Forxiga"[Mesh] OR "Farxiga"[Mesh] OR "(2S,3R,4R,5S,6R)-2-(4-chloro-3-(4-ethoxybenzyl)phenyl)-6- (hydroxymethyl)tetrahydro-2H-pyran-3,4,5-triol"[Mesh] OR "2-(3-(4-ethoxybenzyl)-4-chlorophenyl)-6-hydroxymethyltetrahydro-2H-pyran-3,4,5-triol"[Mesh] OR "BMS 512148"[Mesh] OR "BMS512148"[Mesh] OR "BMS-512148"[Mesh])

AND

("Heart Failure"[Mesh] OR "Heart Failure, Diastolic"[Mesh] OR "Heart Failure, Systolic"[Mesh] OR "Cardiac Failure"[Mesh] OR "Heart Decompensation"[Mesh] OR "Decompensation, Heart"[Mesh] OR "Heart Failure, Right-Sided"[Mesh] OR "Heart Failure, Right Sided"[Mesh] OR "Right-Sided Heart Failure"[Mesh] OR "Right Sided Heart Failure"[Mesh] OR "Myocardial Failure"[Mesh] OR "Congestive Heart Failure"[Mesh] OR "Heart Failure, Congestive"[Mesh] OR "Heart Failure, Left-Sided"[Mesh] OR "Heart Failure, Left Sided"[Mesh] OR "Left-Sided Heart Failure"[Mesh] OR "Left Sided Heart Failure"[Mesh] **OR** “Heart Failure”[tiab] OR “Cardiac Failure”[tiab] OR “Myocardial Failure”[tiab] OR “Heart Decompensation”[tiab] OR “Cardiac Decompensation”[tiab] OR “Myocardial Decompensation”[tiab] OR “Right-Sided Heart Failure”[tiab] OR “Right Sided Heart Failure”[tiab] OR “Congestive Heart Failure”[tiab] OR “Left-Sided Heart Failure”[tiab] OR “Left Sided Heart Failure”[tiab] OR “Heart Incompetence”[tiab] OR “Cardiac Incompetence”[tiab] OR “Myocardial Incompetence”[tiab] OR “Heart Insufficiency”[tiab] OR “Cardiac Insufficiency”[tiab] OR “Myocardial Insufficiency”[tiab] OR “Chronic heart failure”[tiab] OR “Chronic heart insufficiency”[tiab] OR “Chronic heart decompensation”[tiab])

AND

("Randomized Controlled Trial"[Publication Type] OR Randomized Controlled Trial[Mesh] OR "Randomized Controlled Trials as Topic"[Mesh] OR "Controlled Clinical Trial"[Publication Type] OR Controlled Clinical Trial[Mesh] OR "Clinical Trials as Topic"[Mesh] OR "Clinical Trial"[Publication Type] OR Clinical Trial[Mesh] OR Intervention Study[Mesh] OR "Random Allocation"[Mesh] **OR** "Randomized Controlled Trial"[tiab] OR "Randomized Controlled Study"[tiab] OR "Randomized Controlled Trials as Topic"[tiab] OR "Clinical Trial"[tiab] OR Intervention Study[tiab] OR "Random Allocation"[tiab] OR Randomization[tiab] OR "Controlled Clinical Trial"[tiab] OR "Clinical Trials as Topic"[tiab] OR "Clinical Trial as Topic"[tiab])

2) Scopus:

(dapagliflozin OR Forxiga OR "(2S,3R,4R,5S,6R)-2-(4-chloro-3-(4-ethoxybenzyl)phenyl)-6-(hydroxymethyl)tetrahydro-2H-pyran-3,4,5-triol" OR "2-(3-(4-ethoxybenzyl)-4-chlorophenyl)-6-hydroxymethyltetrahydro-2H-pyran-3,4,5-triol" OR "BMS 512148" OR "BMS512148" OR "BMS-512148")

AND

("Heart Failure" OR "Cardiac Failure" OR "Myocardial Failure" OR "Heart Decompensation" OR "Cardiac Decompensation" OR "Myocardial Decompensation" OR "Heart Incompetence" OR "Cardiac Incompetence" OR "Myocardial Incompetence" OR "Heart Insufficiency" OR "Cardiac Insufficiency" OR "Myocardial Insufficiency")

AND

("Randomized Controlled Trial" OR "Randomized Controlled Trials as Topic" OR "Clinical Trial" OR "Intervention Study" OR "Random Allocation" OR Randomization OR "Controlled Clinical Trial" OR "Clinical Trials as Topic" OR "Clinical Trial as Topic" OR "Randomized controlled study")

3) ScienceDirect:

("dapagliflozin" OR "Forxiga" OR "Farxiga") AND ("Heart Failure" OR "cardiac failure" OR "Myocardial failure") AND ("randomized controlled trial" OR "controlled clinical trial" OR "Randomized controlled study")

Figure 1: Risk of bias summary

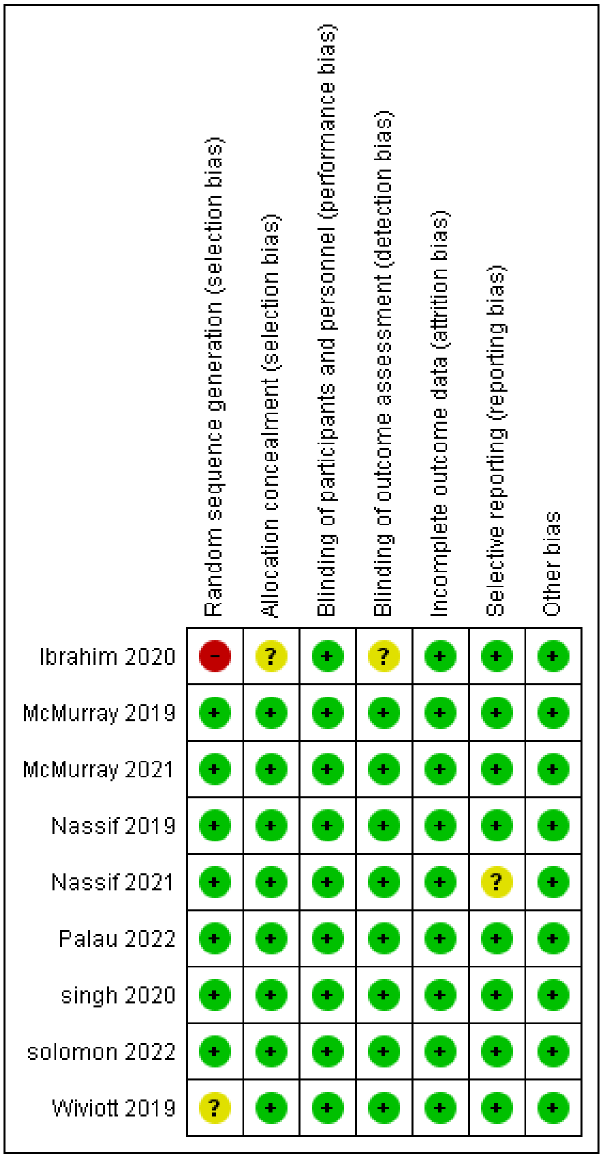

Figure 2: Funnel plot for publication bias assessment

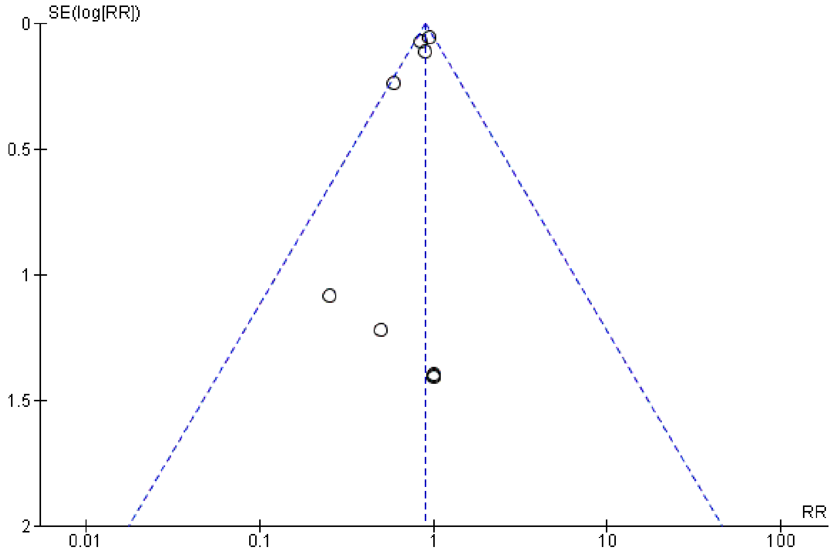

Supplement: Supplementary File. — The supplementary file contains the search strategy and keywords that were used for this study, the risk of bias summary, and the funnel plot for assessment of the publication bias. [file gh-18-1-1258-s1.pdf]
